# Activation of RSK by phosphomimetic substitution in the activation loop is prevented by structural constraints

**DOI:** 10.1038/s41598-019-56937-3

**Published:** 2020-01-17

**Authors:** Desiana Somale, Giovanna Di Nardo, Laura di Blasio, Alberto Puliafito, Marianela Vara-Messler, Giulia Chiaverina, Miriam Palmiero, Valentina Monica, Gianfranco Gilardi, Luca Primo, Paolo Armando Gagliardi

**Affiliations:** 1 Department of Oncology, University of Torino, Turin, 10060 Italy; 2 Candiolo Cancer Institute-FPO IRCCS, Candiolo, 10060 Italy; 3 Department of Life Sciences and Systems Biology, University of Torino, Via Accademia Albertina 13, Torino, Italy; 4 CrisDi, Interdepartmental Center for Crystallography, University of Torino, Via Pietro Giuria 7, Torino, Italy

**Keywords:** Kinases, Molecular modelling

## Abstract

The activation of the majority of AGC kinases is regulated by two phosphorylation events on two conserved serine/threonine residues located on the activation loop and on the hydrophobic motif, respectively. In AGC kinase family, phosphomimetic substitutions with aspartate or glutamate, leading to constitutive activation, have frequently occurred at the hydrophobic motif site. On the contrary, phosphomimetic substitutions in the activation loop are absent across the evolution of AGC kinases. This observation is explained by the failure of aspartate and glutamate to mimic phosphorylatable serine/threonine in this regulatory site. By detailed 3D structural simulations of RSK2 and further biochemical evaluation in cells, we show that the phosphomimetic residue on the activation loop fails to form a critical salt bridge with R114, necessary to reorient the αC-helix and to activate the protein. By a phylogenetic analysis, we point at a possible coevolution of a phosphorylatable activation loop and the presence of a conserved positively charged amino acid on the αC-helix. In sum, our analysis leads to the unfeasibility of phosphomimetic substitution in the activation loop of RSK and, at the same time, highlights the peculiar structural role of activation loop phosphorylation.

## Introduction

The 61 human AGC kinases form a monophyletic group of serine/threonine kinases that preferably phosphorylates residues in close proximity of basic amino acids such as Arg (R) and Lys (K)^1,2^. The kinase domains (KD) of all the AGC kinases share the same tertiary structure, characterized by an amino-terminal small lobe (N-lobe) and a carboxy-terminal large lobe (C-lobe), as originally described for PKA^3^. The two lobes form a pocket that binds one molecule of ATP as phosphate donor during substrate phosphorylation.

**Figure 1:**
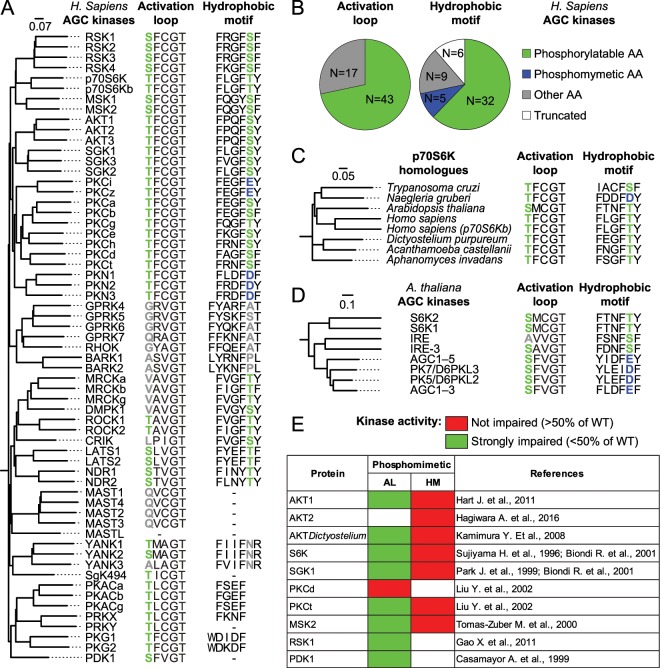
Phylogenetic analysis reveals the absence of phosphomimetic substitutions in the activation loop (AL) of AGC kinases. **(A)** Comparison of AL and hydrophobic motif (HM) sequences across the 61 human AGC kinases. Phosphorylatable residues in AL and HM are represented in green. Phosphomimetic substitutions are represented in blue. Substitutions with neither phosphorylatable nor phosphomimetic amino acids are represented in gray. The scale bar represents the number of amino acid substitutions per site. **(B)** Representation of the proportion of amino acid substitutions of the phosphorylatable residues across human AGC kinases. **(C)** Phylogenetic relationship of *Naegleria gruberi* p70S6K, showing a phosphomimetic substitution in the HM sequence, with p70S6K homologues in other eukaryotic organisms. **(D)** Phylogenetic relationship among some of the *Arabidopsis thaliana* AGC kinases showing phosphomimetic substitution in HM of kinases belonging to AGC1 class. **(E)** Meta-analysis of the effects of artificial phosphomimetic substitutions in different AGC kinases. The results of phosphomimetic substitution on the kinase activity were classified into two groups, one showing a consistent reduction of the kinase activity (residual kinase activity less than 50% of wild type protein), the other showing at least 50% of kinase activity of the wild type protein.

The transition from inactive to active state in AGC kinases is achieved through conformational rearrangements of key structural elements, such as the activation segment and the αC-helix. The activation segment is a sequence of variable length (from 25 aa of PKAa to 43 aa of MAST1) spanning from Asp-Phe-Gly (DFG) to Ala-Pro-Glu (APE) sequences, and including the activation loop (AL) and the P + 1 loop^4^. The DFG sequence is part of the ATP binding site whose orientation defines the active (DFG-in)^3^ and the inactive (DFG-out) states of AGC kinases^5^. The AL contains, in 43 out of 61 AGC kinases (Fig. 1A), a key phosphorylatable site (consensus sequence S/T-x-x-G-T), found to be substrate of 3-Phosphoinositide-dependent protein kinase-1 (PDK1)^6^. The phosphate group added on the AL form a complex set of salt bridges with basic amino acid groups, that in PKA are respectively: R165 in the catalytic loop, H87 in the αC-helix and K189 in the AL, just after the DFG motif^7,8^. By connecting these residues, the phosphorylation of the AL promotes the transition in a more ordered conformation, the stabilization of the two lobes in the closed/active conformation and the assembly of a key hydrophobic core, defined R-spine^9–11^. Crucial event in the transition in the active conformation is the re-orientation of the αC-helix^12^. This event coordinates the formation of key hydrogen bonds between a Glu residue on the αC-helix, a Lys residue in the N-lobe and the phosphate of ATP, and contributes to the assembly of the R-spine^13^.

Moreover, the αC-helix forms, together with the N-lobe, a regulatory hydrophobic pocket. This site mediates the interactions between the N-lobe and the hydrophobic motif (HM)^14^, a sequence (consensus F-x-x-F/Y-S/T-F/Y) localized on the C-terminal tail and present in 53 out of 61 AGC kinases (Fig. 1A). The HM extends from the C-lobe and, wrapping the N-lobe, it inserts two aromatic residues into the hydrophobic pocket^13^. Phosphorylation of AGC kinases on the conserved serine or threonine of the HM plays a dual crucial role in their activation: 1) the phosphorylated HM serves as docking site for the PIF binding pocket of PDK1 which in turn phosphorylates the AL; 2) several AGC kinases (e.g. RSK2, S6K1, AKT1, MSK1 and SGK1) harbor a phosphate binding pocket, next to the hydrophobic pocket, that interacts with its own phosphorylated HM. This interaction contributes, in cooperation with phosphorylated AL, to reorient the αC-helix in the active conformation^15^. Besides the AL and HM, some of the AGC kinases have another phosphorylatable site involved in the regulation of their activation, the turn motif, which is localized in the C-terminal tail, preceding the HM. Once phosphorylated, this site helps the C-tail to wrap the N-lobe and addresses the HM to the hydrophobic pocket site^16^. In summary, the phosphorylation events on the above-mentioned sites are among the major events concurring to AGC kinase activation.

In general, the addition of a phosphate group confers novel chemical properties to different amino acids, above all serine (Ser), threonine (Thr) and tyrosine (Tyr)^17^. At the intracellular pH, the phosphate group is only partially deprotonated and −1 and −2 charged species coexist. Thanks to these negative charges, phosphate groups can act as donors for salt bridges with positively charged amino acids, such as arginine (Arg) and lysine (Lys). Moreover, both protonated and deprotonated phosphate oxygens can form hydrogen bonds with different amino acids^18^. Phosphorylation can affect the activity and the function of proteins in different ways: (1) by favoring the disordered-ordered transitions; (2) by allosteric regulation at the level of tertiary and quaternary structures; (3) by changing the recognition properties of protein binding sites; (4) by regulating post-translational modifications^19^.

The carboxyl group of aspartate (Asp) and glutamate (Glu) is also deprotonated at intracellular pH and can mimic the phosphate group, especially for the −1 charged species^20^. Therefore, for more than 30 years, protein phosphorylation has been artificially mimicked in the lab by phosphomimetic substitutions of phosphorylatable sites with Asp and Glu^21^. Remarkably, such substitution is frequently found throughout the evolution of eukaryotes. Moreover, the opposite process has also been shown to occur: phosphorylatable residues can emerge by mutation of preexisting phosphomimetic amino acids^20^.

Here, we focus our attention on the unusual low frequency of phosphomimetic substitution on the AL, compared to the HM, in the evolution of AGC kinases. To investigate the reasons of this AL distinctive feature we performed biochemical, mutational and *in silico* studies on the AGC kinase RSK2. Whereas the phosphorylated AL interacts with three key residues, Arg114, Arg192 and Lys216 in RSK2, the phosphomimetic amino acid substituted to the phosphorylatable residue in the AL binds only to Arg192 and Lys216. The inability of the phosphomimetic substitution to correctly interact with all three residues compromises the molecular conformational transitions required for the activation of the enzymatic activity and explains the failure of the phosphomimetic substitution. Combining this result with a phylogenetic analysis, we highlight the unfeasibility of the phosphomimetic substitution on the AL of AGC kinases.

## Results

### Absence of phosphomimetic substitution in the AL during eukaryotic evolution

Phosphorylation on Ser or Thr in the activation loop (AL) and in the hydrophobic motif (HM) of AGC kinases represents a critical event leading to their activation. To investigate whether these key serine/threonine residues have been replaced by phosphomimetic amino acids (Asp or Glu) during protein kinase evolution, we aligned the protein sequences of all human AGC kinases. 60 out of 61 AGC kinases show a conserved AL sequence and 53 out of 61 AGC kinases show a HM sequence, although in some kinases, including PKA, there is only a truncated form of it (Fig. 1A). The comparison of AL sequences highlights that a phosphorylatable residue (Ser or Thr) is present in the majority of AGC kinases. In the other kinases, the AL phosphorylatable residue has been substituted with a non-charged amino acid and no phosphomimetic substitution is present (Fig. 1A).

The phosphorylatable residue in the HM is present in 32 AGC kinases. In the other cases the HM motif is truncated, harboring other amino acids or a phosphomimetic substitution. Specifically, in two isoforms of PKC (PKCi and PKCz) and three isoforms of PKN (PKN1, PKN2 and PKN3) the phosphorylatable residue is replaced by Glu and Asp, respectively (Fig. 1A). The number of replacements of Ser or Thr by non-charged amino acids in the AL and by phosphomimetic amino acids in the HM suggests a different propensity for amino acid substitutions between these two critical sites (Fig. 1B). Phosphomimetic residues also appeared in the HM outside of the human kinome. *Naegleria gruberi*, a unicellular eukaryote, has evolved a phosphomimetic substitution in the HM of the orthologue of human p70S6K (Fig. 1C). Likewise, plant AGC kinases, belonging to class AGC1 (e.g. AGC1–3, AGC1–5, PK5/D6PKL2 and PK7/D6PKL3)^22^, have evolved phosphomimetic substitutions in the HM. Analogously with human AGC kinases, phosphomimetic substitutions are not observed in the AL (Fig. 1D).

The lack of phosphomimetic substitutions in the AL raises the question whether the negative charges of Asp and Glu are actually able to mimic the negative charges of phosphorylated amino acids in the AL. Since phosphomimetic substitution is widely used as a tool for biochemical studies, we carefully analyzed published data reporting the effects of such substitutions on the kinase activity (Fig. 1E). In the analyzed literature, the phosphomimetic substitutions in AL resulted in a strong reduction of the kinase activity in AKT1^23^, *Dictyostelium discoideum* AKT^24^, MSK2^25^, PDK1^26^, PKA^7,27^, PKCt^28^, p70S6K^29^, SGK1^30^ and RSK1^31^. The only exception is PKCd where a phosphomimetic substitution in the AL resulted in a modest increase of the kinase activity^28^. This is in agreement with a previous report showing that phosphorylation of PKCd AL was not required for its kinase activity^32^.

Contrariwise, phosphomimetic substitutions in the HM do not hamper or promote the kinase activity of AKT1^23^, AKT2^33^, *Dictyostelium discoideum* AKT^24^, MSK2^25^, PKCt^28^, p70S6K and SGK1^34^. This analysis suggests that phosphomimetic residue in the AL are unable to correctly simulate the phosphorylation of Ser or Thr.

### Correlation between the phosphorylation status of the AL and the kinase activity in RSK

To elucidate why the phosphomimetic substitution in the AL is functionally inactive, we experimentally studied this amino acid substitution in RSK protein kinases. RSKs are composed by two kinase domains: a N-terminal kinase domain (NTKD), belonging to AGC kinase family, and a C-terminal kinase domain (CTKD), belonging to the CaMK (Calcium/calmodulin-dependent protein kinase) family^35–37^. The CTKD is phosphorylated and activated by ERK that binds RSK on a specific docking site localized in the C-terminal tail^38^. The activation of NTKD is instead a classic two-steps process: first the active CTKD phosphorylates the HM in Ser380 for RSK1 and Ser386 for RSK2, then 3-phosphoinositide-dependent kinase 1 (PDK1) binds to the phosphorylated HM and phosphorylates the AL on Ser221 for RSK1 and Ser227 for RSK2^35,39,40^.

**Figure 2:**
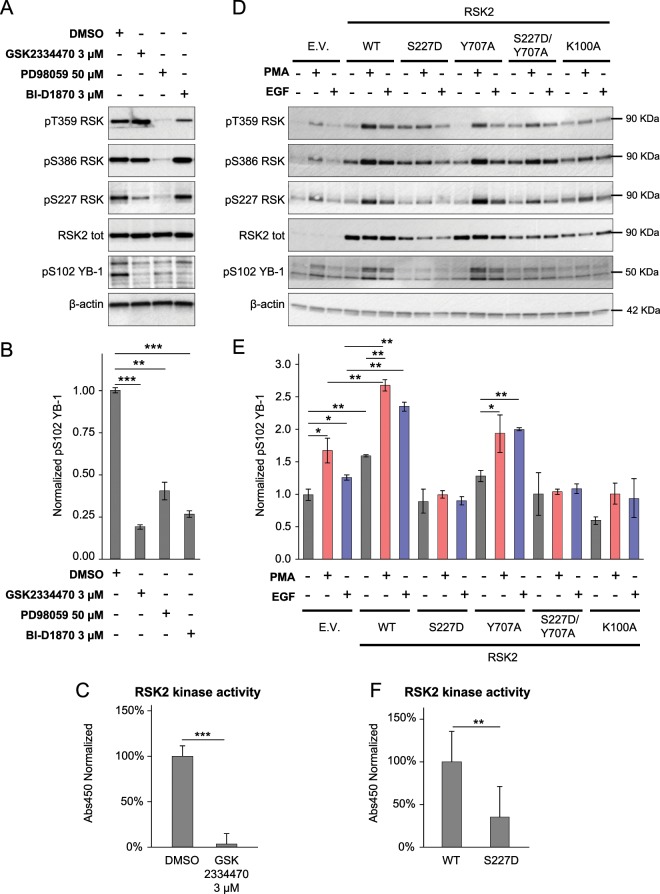
RSK2 kinase activity is impaired by phosphomimetic substitution of Ser227 in the NTKD. (**A)** HeLa cells were transfected with a lentiviral vector containing the RSK2 coding sequence. These cells were starved overnight with normal culture medium without FCS and with or without the addition of the specific PDK1 inhibition GSK2334470 at 3 μM or the MEK inhibitor PD98059 at 50 μM or the RSK inhibitor BI-D1870 at 3 μM. Then, HeLa cells were stimulated with EGF (10 ng/ml) for 15 min and lysed. Western blot analysis was performed using the indicated primary antibodies. β-actin was used as a loading control. **(B)** Quantification of the YB-1 phosphorylation on the serine 102 compared to β-actin level. Data were plotted as the mean ± s.d. of three independent experiments. **(C)** 293T cells were transfected with pDEST27-RSK2WT plasmid. Then, cells were treated or not with the PDK1 inhibitor GSK2334470 (3 μM). After that, cells were lysed on ice. GST-RSK2WT was isolated from 293T cells extracts and assayed for the phosphorylation of a specific synthetic peptide as substrate. **(D)** HeLa cells were transfected with a lentiviral vector containing the wild type or mutated (S227D, Y707A, S227D/Y707A and K100A) RSK2 coding sequence or an empty vector (E.V.). Then, cells were serum-starved overnight and stimulated or not with PMA (60 ng/ml) or with EGF (10 ng/ml) for 5 min and lysed. Western blot staining was performed using the indicated primary antibodies. β-actin was used as a loading control. **(E)** Quantification of the YB-1 phosphorylation on the serine 102 compared to β-actin level. Data were plotted as the mean ± s.d. of three independent experiments. **(F)** 293T cells were transfected with pDEST27-RSK2WT or pDEST27-RSK2S227D plasmids and cultured without FCS (serum free, sf). Then, cells were lysed on ice. GST-RSK2WT and GST-RSK2S227D were isolated from 293T extracts and assayed for the phosphorylation of a peptide substrate.

We evaluated the phosphorylation of a RSK substrate as well as of RSK internal regulatory sites in RSK2 overexpressing HeLa cells stimulated with EGF (10 ng/ml) and in the presence of PDK1 inhibitor GSK2334470 (3 μM), MEK inhibitor PD98059 (50 µM) or RSK inhibitor BI-D1870 (3 µM) (Fig. 2A). The phosphorylation status was evaluated by immunoblot using antibodies recognizing the phosphorylated forms of Thr359 (a critical phosphorylatable residue substrate of ERK), Ser386 and Ser227 of RSK2. The activity of RSK2 was instead evaluated on the amount of the phosphorylated RSK2 substrate Y-box binding protein-1 (YB-1)^41,42^ (Fig. 2A,B).

As previously reported^40,43–45^, RSK2 is characterized by high basal levels of phosphorylation on all its internal residues (Thr359, Ser386 and Ser227), as well as at the level of phosphorylation of its substrate YB-1 on Ser102 (Fig. 2A,B). In presence of the PDK1 inhibitor GSK2334470, both Ser227 and YB-1 phosphorylation were dramatically reduced (Fig. 2A,B). In the presence of the MEK inhibitor PD98059, all the phosphorylation of all RSK2 residues and of YB-1 were drastically reduced confirming the importance of the regulation by ERK (Fig. 2A,B). As expected, the inhibition of NTKD with BI-D1870 reduces only the downstream phosphorylation of YB-1 and not the one of the regulatory sites on RSK2 itself (Fig. 2A,B). We also performed an *in vitro* kinase assay to test the biochemical activity of recombinant RSK2 purified from cells treated or not with the PDK1 inhibitor GSK2334470 (3 μM). The inhibition of PDK1 determined a complete inactivation of RSK2 *in vitro* enzymatic activity (Fig. 2C).

### Substitution of the phosphorylatable residue in the AL with Asp abrogates RSK kinase activity in cells and *in vitro*

To investigate the inability of the phosphomimetic substitution to recapitulate the phosphorylation of the AL, we generated a mutant of RSK2 with a Ser (S) substituted with an Asp (D) in position 227 (S227D). As a negative control, we used a kinase-dead mutant of the NTKD, i.e. K100A, where the mutation is located in the ATP-binding site in the NTKD, which leads to inactivation of the kinase activity of the protein^37,46^. As a positive control, we used a constitutively active mutant of RSK2, with the mutation Y707A located in the C-terminal tail that overcomes the need of phosphorylation by ERK^47^. As a further control, we generated a double mutant of RSK2, S227D/Y707A, to understand if the phosphomimetic substitution in the AL is able to compromise the NTKD kinase activity even in presence of constitutive upstream signal.

The phosphorylation levels of RSK2 at Thr359, Ser386 and Ser227 were increased by EGF or PMA acute stimulation in both cells transduced with the empty vector and overexpressing RSK2, indicating full activation of NTKD (Fig. 2D). As expected, the phosphorylation of YB-1 increased upon stimulation with both EGF and PMA (Fig. 2D,E). Cells overexpressing S227D mutant of RSK2 failed to display increased YB-1 phosphorylation in response to both PMA and EGF. Moreover, both the phosphorylations of Thr359 and Ser386 were compromised in S227D mutant of RSK2 (Fig. 2D,E), suggesting the existence of an allosteric linkage between active NTKD and the C-terminal docking site for its activator ERK, as previously observed with a kinase inactive mutant^48^. The lack of activity of S227D is comparable to that of the kinase dead K100A mutant (Fig. 2D,E). This observation is in agreement with the original discovery that a phosphomimetic mutant in the AL of PKA has reduced kinase activity compared to the wild type protein^7^, and with the results in other AGC kinases (Fig. 1E).

The expression of RSK2 Y707A promoted the phosphorylation of YB-1 upon stimulation with both EGF and PMA (Fig. 2D,E). According to the original publication^47^, this mutant shows the same kinase activity as RSK2 WT in cells. Despite this mutant may disconnect the feedback regulation of the ERK docking site by the NTKD, the presence of S227D phosphomimetic mutation overcomes such effect and abrogates RSK2 kinase activity (Fig. 2D,E).

We then verified the kinase activity of S227D mutant of RSK2 in a cell-free system. Our results indicate that the kinase activity of this mutant is significantly reduced compared to the wild type protein, even though not absent (Fig. 2F). This observation is also supported by a recent report where the mutant RSK2 S227E failed to activate RhoA GTPase through its compromised kinase activity on LARG, a GEF for RhoA. In the same report, the phosphomimetic mutation on Ser386 (the HM) instead potentiates this pathway^49^.

### Structural interactions between the phosphorylated AL and three positively charged residues define the active conformation of AGC kinases

Since phosphomimetic mutants have been widely exploited for scientific research purposes, the compromised kinase activity of RSK2 S227D mutant prompted us to investigate the underlying molecular reasons of this failure. Indeed, while the low kinase activity of phosphomimetic mutant in the AL has already been described in other kinases of the AGC kinase family, the underlying mechanism has never been further explored. However, a study on PAK1 (p21-activated kinase 1), which does not belong to AGC kinase family but harbors an AL phosphorylated by PDK1^50^, showed that the phosphomimetic mutant (T423E) determines failure of kinase activation. This is due by the inability of such phosphomimetic substitution to recapitulate the bonds formed by the phosphorylated Thr^51^.

**Figure 3:**
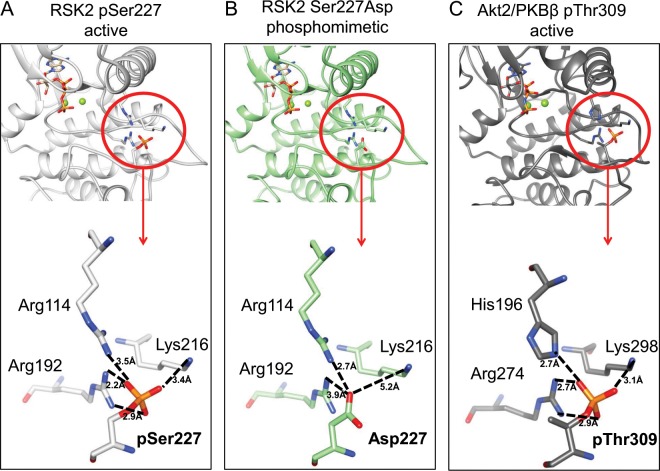
Crystal structures of active RSK2 and Akt2/PKBβ show the interaction between the phosphate group on the AL and three positively charged amino acids. (**A)** The crystal structure of N-terminal wild type RSK2 kinase in complex with 2-amino-7-substituted benzoxazole (PDB ID 4NW5) shows the interactions between pSer227 and other three positively charged amino acids: Arg114, Arg192 and Lys216. **(B)** In the same structure of RSK2, pS227 was substituted with an Asp. **(C)** Structure of the active AL in Akt2/PKBβ (PDB ID 1O6K), where Thr309 interacts with Hys196, Arg274 and Lys298.

To understand if this situation also occurs in AGC kinases, we analyzed a crystal structure of the active N-terminal RSK2 kinase showing the lowest root-mean-square deviation (*RMSD*) value (0.954 Å) (PDB ID 4NW5) in comparison with the active Akt2 (PDB ID 1O6K). The starting protein showed that pSer227 is part of a hydrogen bond network which involves Arg114, Arg192 and Lys216 in RSK2 (Fig. 3A) and His196, Arg274 and Lys298 in Akt2 (Fig. 3C). Arg114 in RSK2 and His196 in Akt2 protrude from the second turn of the αC-helix toward the AL. Arg192 in RSK2 and Arg274 in Akt2 are localized just after the β6 strand, between the long αE-helix and β7 strand in the C-lobe. Lys216 in RSK2 and Lys298 in Akt2 are in the β9 strand, just after the DFG motif of the ATP binding site. Both RSK2 and Akt2 follow indeed the prototypical structure of PKA, solved in 1991, where the pT197 in the AL loop was shown to interact with Arg165, His87 and Lys189^3,7^, corresponding to RSK2 residues Arg192, Arg114 and Lys216 respectively^36^.

The network formed by the phosphate group of the AL and positively charged amino acids is crucial to obtain a well-structured AL. This conformation is characterized by specific orientation of the αC-helix, anchored to the phosphate on Ser227 by means of Arg114, that carries a conserved acidic residue forming an ion pair with a conserved lysine in the active site, enabling cofactor binding, as previously shown for PAKs^52^. When Ser227 is substituted with Asp, even though the structure is superimposable with pSer227, the strength of interactions is decreased and the N atom of Lys216 side chain is 5.2 Å away from the O atom of Asp side chain (Fig. 3B). Thus, this residue does not participate in the stabilization of the loop. Moreover, Asp227 can potentially interact with only one of its oxygen atoms with Arg192 even if the distance Asp227-Arg192 is larger (3.9 Å) compared to that of pSer227-Arg192 (2.2 Å), where two interactions are predicted to form. Thus, the overall structural stability of the loop seems to be lower when Asp is present in position 227 (Fig. 3B).

### Computational modeling of RSK2 structure with phosphomimetic substitution in the AL reveals a transition toward an inactive conformation

In order to investigate the effect of the phosphomimetic substitution on the dynamics of the AL and of the entire protein, we performed molecular dynamics (MD) simulations of the active wild type RSK2 (with the phosphorylated Ser227) and of RSK2 harboring the mutation S227D. As a readout of MD simulation, we used the Root-Mean-Square Deviation (*RMSD*) and the Root-Mean-Square Fluctuations (*RMSF*).

**Figure 4:**
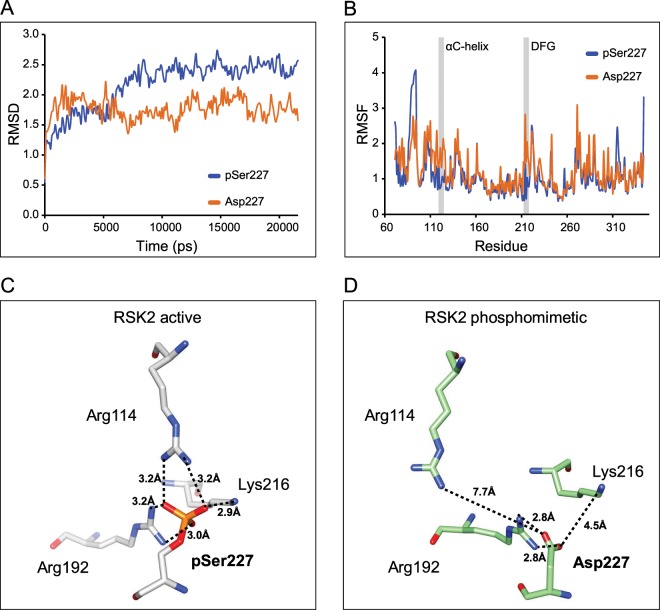
Molecular dynamics simulation of RSK2 in the active and phosphomimetic forms. **(A)** The *RMSD* time traces during the MD simulation show a rapid increase within 2 ns. Then, pSer227 simulation shows a slow increase in the *RMSD*, while Asp227 simulation presents a plateau. **(B)** The *RMSF* profiles show that αC-helix (residues from 119 to 124) and the DFG region (from 221 to 218) were more flexible in the mutant RSK2 S227D than in the active wild type protein (pSer227). **(C)** The crystal structures of the activation loop of phosphorylated wild type RSK2 and of **(D)** the phosphomimetic form obtained after MD simulation of PDB ID 4NW5. pSer227 after MD simulation maintains all the three bonds shown in the crystal structure with Arg114, Lys216 and Arg192. On the contrary, Asp227 in the phosphomimetic protein interacts only with Lys216 and Arg192.

In our MD simulation, the *RMSD* profile showed a different behavior for pSer227 and Asp227 over time. In both cases, a first rapid increase in the *RMSD* within 2 ns was observed. After this time, the Asp227 simulation reached a stationary state, whereas the pSer227 took longer to reach a plateau (Fig. 4A). The *RMSF* profiles as a function of the residue position also showed that the region 119–124 (corresponding to αC-helix) and 212–218 (corresponding to the loop carrying the DFG motif) are more flexible for the Asp227 mutant rather than the pSer227 one (Fig. 4B). The analysis of the AL in the average structure of the MD simulation as well as in the final structure after 20 ns of the simulation showed that the H-bonds network involving pSer227 was still present with distances between the O atoms of the phospho-Ser and the N atoms of Lys and Arg residues ranging from 2.9 to 3.2 Å (Fig. 4C). On the contrary, when the S227D mutation was present, Arg114 was free to move away triggering a larger shift in the αC-helix, compared to that induced after the Ser phosphorylation (Fig. 4D). The shift in the αC-helix was accompanied in both simulations by the propensity of this element to unfold starting from the C-terminal. Therefore, the MD simulation results allowed us to conclude that, in the case of the phosphomimetic mutant, the decreased stabilization of the AL and the loss of the interaction with the Arg residue present on αC-helix play a key role in the propensity of the protein conformation to change from the inactive state to the active state.

In summary, our MD simulation supports the hypothesis that the presence of a phosphate group on the AL of RSK2 is critical for its activation. Consequently, the mere presence of different negatively charged residues in place of the phosphate group is unable to cause the same conformational rearrangements necessary to determine kinase activation. This model could be likely extended to other members of the AGC kinase family.

### Mutation of Arg114 in the αC-helix of RSK2 phenocopies the substitution with Asp in the AL

**Figure 5:**
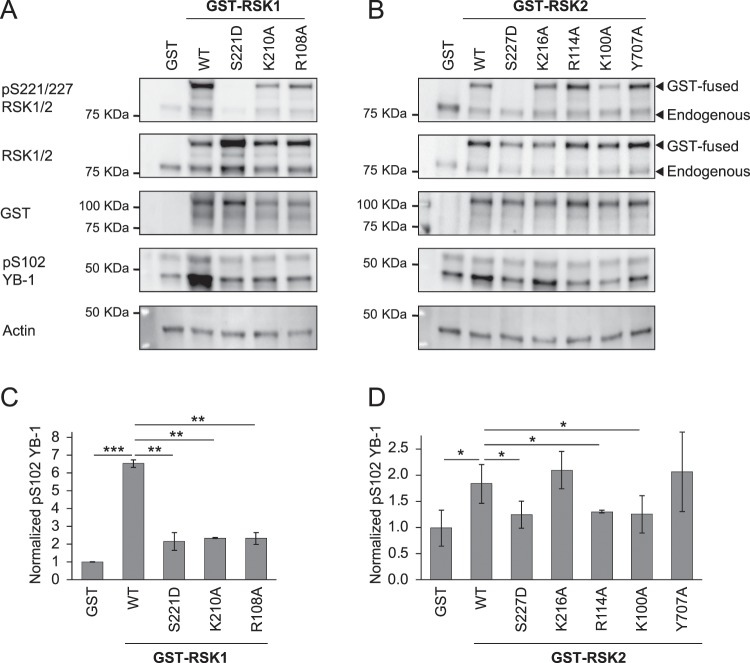
RSK1 and RSK2 kinase activity is impaired by the substitution of Arg108 in RSK1 and Arg114 in RSK2. (**A,B)** HeLa cells were transfected with pDEST27 plasmid coding for wild type RSK1 and RSK2, the phosphomimetic mutant (S221D for RSK1 and S227D for RSK2), the mutants in the amino acids shown to interact with the AL phosphate (K210A and R108A for RSK1, K216A and R114A for RSK2), the kinase dead mutant (K100A in RSK2) and the constitutively active mutant (Y707A) fused with GST. As a control, we used a pDEST27 expressing GST only. Such cells were kept overnight with normal culture medium. Then, HeLa cells were starved for 3 h with normal culture medium without FCS. After that, these cells were stimulated with EGF (10 ng/ml) for 15 min and lysed. Western blot analysis was performed using the indicated primary antibodies. β-actin was used as a loading control. **(C-D)** Quantification of the YB-1 phosphorylation on the Ser 102 normalized on β-actin level. Data were plotted as the mean ± s.d of three independent experiments.

MD simulations showed that, compared with pSer227, the side chain of Asp227 in phosphomimetic RSK2 loses the interaction with Arg114. To understand if this interaction is critically important in the induction of the kinase activity, we generated the mutant R114A and, as an additional control, we performed the same experiment with RSK1 generating the mutant R108A (Fig. 5A,B). Moreover, since the distance between Asp227 and Lys216 is increased compared to the pSer227 protein, we generated a K216A mutant of RSK2 and, in this case as well, we mutated the corresponding residue in RSK1 producing the mutant K210A (Fig. 5A,B). As positive and negative controls, we used the constitutive active mutant of RSK2 (Y707A) and the kinase dead mutant in the N-terminal kinase domain of RSK2 (K100A) (Fig. 5A,B).

As expected, overexpression of both RSK1 and RSK2 GST-fusion proteins in HeLa cells is able to significantly increase the phosphorylation of YB-1 (Fig. 5A–D). This increase in YB-1 phosphorylation is compromised by the phosphomimetic substitution in Ser221 for RSK1 and Ser227 in RSK2 (Fig. 5A–D). Similarly, the substitution of Arg108 in RSK1 and Arg114 in RSK2 markedly hampered the increase of YB-1 phosphorylation. In RSK2, YB-1 phosphorylation in R114A expressing cells has the same low levels of those expressing the kinase dead mutant K110A. Such results highlight the crucial role of this αC-helix residue for the activation of kinase activity (Fig. 5A–D). Conversely, the mutation of Lys216 in RSK2 does not compromise the kinase activity on YB-1, while the same mutation in RSK1 (K210A) is able to reduce it (Fig. 5A–D). Even though we cannot explain this discrepancy, the behavior of RSK2 mutant suggests that Lys216 residue is less important for the activation of the N-terminal kinase.

### Phylogenetic analysis reveals a correlation between the presence of a phosphorylatable AL and the presence of a positively charged amino acid on the αC-helix

Our MD simulations pointed at a crucial role of the bond between phosphate on the AL and Arg114 on the αC-helix for the correct structural rearrangement in the active state of RSK2. Our experimental data further confirmed that Arg114 is critically required for the activation of the enzymatic activity of the protein. To understand if this observation could be applied to other members of the AGC family, we decided to investigate the conservation of the three positively charged amino acids interacting with the phosphate of the AL.

**Figure 6:**
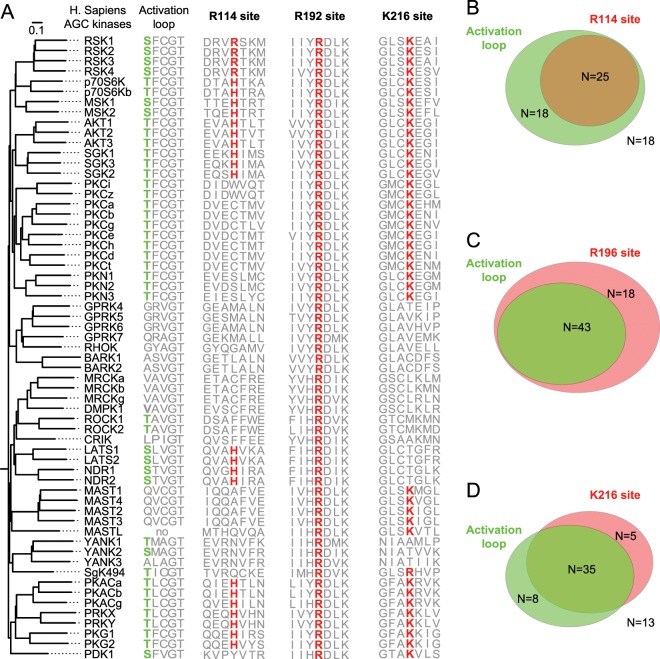
The presence of a phosphorylatable AL correlates with the presence of RSK2 Arg114 and Lys216 homologues in the AGC kinase family. (**A**) Phylogenetic tree of AGC kinases, according to the alignment of the kinase domain only, showing the sequences of the AL, Arg114 (R114), Arg192 (R192) and Lys216 (K216) in RSK2 and their homologues in the other member of AGC kinases. The presence of a phosphorylatable AL is indicated in green, whereas the presence of a positively charged amino acid in Arg114, Arg192 and Lys216 in the same position of RSK2 is indicated in red. **(B)** Euler diagram showing the overlap between the presence of a phosphorylatable AL and the presence of Arg114 site. 25 out of 43 AGC kinases showing the phosphorylatable AL display a positively charged amino acid in the Arg114 site. **(C)** Euler diagram showing that the Arg192 site is present in all the AGC kinases, even in those lacking a phosphorylatable AL, suggesting an additional role independent from the interaction with AL phosphate. **(D)** Euler diagram showing the intersection between AGC kinases having a phosphorylatable AL and the positively charged amino acid in the Lys216 site.

As shown in Figs. 1A and 6A, the AL harbors a phosphorylatable Ser or Thr residue in 43 out of 61 AGC kinases. Interestingly, the Arg114 site is only present in the kinases having a phosphorylatable AL, while it is absent in the kinases lacking the conserved Ser or Thr in the AL (Fig. 6A). Therefore, the kinases having the Arg114 represent a subset of the kinases with the phosphorylatable AL (25 out of 43 kinases) (Fig. 6B). The fact that 18 of these 43 kinases lack of the Arg114 site may suggest that the function of Arg114 in the molecular rearrangement of the αC-helix after AL phosphorylation has been substituted by another positively charged residue or that such kinase do not depend on AL phosphorylation for activation, like PKCd. The Arg192 site is instead conserved in all the 61 AGC human kinases, including those lacking the phosphorylatable residue on the AL (Fig. 6A,C). The widespread presence of this residue across the whole AGC family may indicate that it has a further role in the functionality of the kinase domain beyond the ability to bind the phosphate on the AL. Finally, the Lys216 site is present in all the AGC kinases that rely on the phosphorylation of the AL, including the subfamilies of RSK, S6K, MSK, AKT, SGK, PKC, PKN, PKA, PRK, PKG and PDK1 (Fig. 6A). Moreover, it is absent in the kinases that lack the phosphorylatable AL or that do not depend on the phosphorylation of this residue for the activation, such as GPRK, BARK, DMPK, LATS/NDR, and YANK subfamilies with the exception of MAST subfamily (Fig. 6A,D).The correlation between the presence of a phosphorylatable AL and Lys216 site points at a possible coevolution of the two sites. The conservation of the Lys216 site may be explained by its role in the phosphotransfer activity of AGC kinases by binding the phosphate of an ATP molecule, as it was previously proposed^36^. However, according to our observation a K216A mutant is insufficient to abrogate substrate phosphorylation by RSK2 (Fig. 5B,D), raising doubts on the role of this site for the kinase activity.

In summary, the presence of the Arg114site in most of the AGC kinases having a phosphorylatable AL suggests a key role of the salt bonds established between the phosphate on the AL and the positively charged side chain of Arg114 site for the full enzymatic activation of AGC kinases. Moreover, it explains why the phosphomimetic substitution, unable to bind to the Arg114 site, has experimentally failed in several of the AGC kinases (Fig. 1E).

## Discussion

A commonly accepted hypothesis suggests that phosphorylatable sites in proteins evolved from preexisting acidic amino acids^20^. According to this hypothesis, the constitutive salt bridge between the negative charge of an acidic amino acid and the positively charged residues evolved in a conditional bond between a phosphate and positively charged amino acids, offering the unique opportunity to control protein conformation by a rather simple biochemical reaction^17,53^. As a consequence of the explosion of the number of sequenced genomes, several events of bidirectional exchange between acidic amino acids and phosphorylatable residues have been reported and further sustain this hypothesis^20^.

By focusing on a monophyletic group of protein kinases, the AGC kinases, we compared the two main regulatory phosphorylatable sites: the hydrophobic motif (HM) and the activation loop (AL). The HM is a prototypical example of the interchangeability between acidic amino acids and phosphorylatable residues, both found in different AGC paralogues and orthologues (Fig. 1). This site has a docking function for PDK1 that in turn phosphorylates the AL^15^. The exchange between acidic amino acids and phosphorylatable residues and vice versa has the power to make the interaction with PDK1 either constitutive or conditional respectively.

Compared to the HM phosphorylation site, the AL one is very different: (1) there is no trace of interchangeability between acidic amino acids and phosphorylatable residues, neither in AGC kinase orthologues nor in paralogues; (2) experiments of phosphomimetic substitutions determine the loss of kinase activation; (3) MD simulations showed that the phosphate on the AL forms a complex interaction with three spatially distant positively charged amino acid that cannot be mimicked by a negatively charged amino acid; (4) the interactions of the phosphate on the AL with these three amino acids are necessary to allow the conformational rearrangements required for the activation of the protein; (5) from a phylogenetic point of view the existence of basic amino acids in the αC-helix and in the β9 strand is interconnected with the existence of a phosphorylatable AL.

Such irreversibility of phosphorylatable to phosphomimetic conversion in the AL might be explained by the long evolutionary path of this regulatory site in AGC kinases. It is well known, in fact, that the reversibility evolution path decreases with the time passed after an amino acid substitution due to the accumulation of other additional substitutions in the same protein. After a long evolutionary period, the reversion of the initial substitution would indeed find a structural situation that is completely different from the original one, and therefore the restoration of the original function of that amino acid would be impossible^54^. It is conceivable therefore that AGC kinases during their history evolved a strict dependence on the presence of a phosphate on the AL, as neither a phosphomimetic amino acid nor other residues are able to recapitulate the complex set of interactions orchestrated by the phosphate group on the AL.

However, evolutionary paths have followed an alternative way to elude the dependence on AL phosphorylation by simply eliminating the AL role in kinase activation. This is the case of the seven members of the Rho-activated protein kinases that lack a regulation mediated by the phosphorylation of AL: in ROCK1 and ROCK2 the phosphorylatable site is present but its phosphorylation does not play any role in kinase activation; in the other five proteins this site is absent. In this family, activation is achieved after protein dimerization, which determines kinase rearrangement in the active conformation, including the stabilization of the αC-helix^13,55^. Similarly, GPRKs lack a phosphorylatable residue in the AL and do not depend on the AL for their activation. The function of this site has instead been substituted by the binding with G-Protein Coupled Receptors (GPCRs), their upstream activators, capable of determining the transition in the active conformation^13^.

Another possible explanation of the different propensity for an effective phosphomimetic substitution in the AL and HM could be found on the different function of the phosphorylation on such sites. The phosphorylation on the AL is mainly involved in disordered/ordered transition and allosteric regulation in the tertiary structure. On the contrary the phosphorylation on the HM modifies the protein binding properties of such sites and is involved in the stabilization of the αC-helix or oligomerization in Rho-associated kinases. It is conceivable that the functions of disordered/ordered transition and allosteric regulation associated with the AL phosphorylation require a more complex set of interactions compared to the HM and explains the lower propensity to phosphomimetic substitution of the first site.

The irreversibility of phosphomimetic substitution in the AL has also a strong implication in biochemical experimentation. Since AL constitutive phosphorylation cannot be mimicked by a phosphomimetic amino acid, it is not possible to separate the activation of AGC kinases from the phosphorylation of their AL by the upstream kinase PDK1. Therefore, research on PDK1-mediated activation of downstream kinases by phosphorylation on the AL has to rely exclusively on loss of function approaches, such as the substitution of the AL phosphorylatable residue in Ala or PDK1 inhibition, silencing or knock-out.

In summary, we have investigated the effects of amino acid substitutions in the AL in RSK, highlighting the structural and evolutionary reasons of AGC kinases’ addiction on the AL phosphorylation.

## Material and Methods

### Cell cultures

Cell lines were handled using standard cell culture conditions. HeLa were obtained from the ATCC resource center and 293T cell lines were cultured in DMEM medium supplemented with 10% FBS (Gibco), 200 U/ml of penicillin, and 200 μg/ml streptomycin (Sigma-Aldrich). All experiments were performed on cell lines that had been passed for <3 months after thawing. Transient gene expression in HeLa was obtained by using Lipofectamin (Thermo-Fisher scientific).

### Chemical compounds

PDK1 inhibitor GSK2334470, MEK inhibitor PD98059 and RSK inhibitor BI-D1870 were purchased from Sigma-Aldrich and dissolved in DMSO to a final concentration of 10 mM. The obtained solutions were aliquoted and stored at −80 °C.

### Plasmid constructs

To mutate RSK1 and RSK2, the following primers were designed:

RSK1S221D FW 5′-CCACGAGAAGAAGGCCTATGATTTCTGCGGGACAGTGG-3′;

RSK1 S221D RE 5′-CCACTGTCCCGCAGAAATCATAGGCCTTCTTCTCGTGG-3′;

RSK1 K210A FW 5′-CTGACTTTGGCCTGAGCGCAGAGGCCATTGACCACGA-3′;

RSK1 K210A RE 5′-TCGTGGTCAATGGCCTCTGCGCTCAGGCCAAAGTCAG-3′;

RSK1 R108A FW 5′-AAGTACGTGACCGCGTCGCGACCAAGATGGAGAGAGA-3′;

RSK1 R108A RE 5’-TCTCTCTCCATCTTGGTCGCGACGCGGTCACGTACTT-3’;

RSK2 S227D FW 5′-CCATGAAAAGAAGGCATATGATTTTTGTGGAACTGTGG-3′;

RSK2 S227D RE 5′-CCACAGTTCCACAAAAATCATATGCCTTCTTTTCATGG-3′;

RSK2 K216A FW 5′-CAGATTTCGGCCTAAGTGCGGAGTCTATTGACCATGA-3′;

RSK2 K216A RE 5′-TCATGGTCAATAGACTCCGCACTTAGGCCGAAATCTG-3′;

RSK2 R114A FW 5′-AAGTTCGAGACCGAGTTGCGACAAAAATGGAACGTGA-3′;

RSK2 R114A RE 5′-TCACGTTCCATTTTTGTCGCAACTCGGTCTCGAACTT-3′.

RSK2K100A and RSK2Y707A was instead provided by Professor Ramos JW^56^.

RSK2WT, RSK2S227D, RSK2K100A, RSK2Y707A and RSK2S227D/Y707A mutants were cloned into pCCL.sin.cPPT.polyA.CTE.eGFP.minhCMV.hPGK.Wpre lentiviral plasmid^57^ to obtain their overexpression in HeLa cells.

All mutants (RSK1WT, RSK1R108A, RSK1K210A, RSK1S221D, RSK2WT, RSK2K100A, RSK2R114A, RSK2K216A, RSK2S227D, and RSK2Y707A) were cloned in pDONR/zeo using Gateway BP Clonase (Thermo Fisher Scientific). These constructs were then transferred through Gateway LR Clonase in the pDEST27 (GST-N-Term Tag, Thermo Fisher Scientific) to obtain fusion proteins.

### Phylogenetic analyses

The phylogenetic tree was obtained from sequence alignment of the kinase domains of human AGC kinases from kinome.com project^1^. Sequence alignment was performed by AlignX (VectorNTI 11) using BLOSUM62 similarity matrix. The guide tree was built using the Neighbor Joining method (NJ) developed by Saitou and Nei^56^. The phylogenetic tree was generated with FigTree v1.4.2 software.

### Western blot

In order to verify RSK2 regulation and activation, HeLa cells overexpressing RSK2WT were treated with following conditions: 1) SF medium overnight, followed by a 15 min stimulation with EGF (10 ng/ml); 2) SF medium supplemented with the PDK1 inhibitor GSK2334470 (3 μM) overnight, followed by a 15 min stimulation with EGF (10 ng/ml); 3) SF medium supplemented with the MEK inhibitor PD98059 (50 μM) overnight, followed by a 15 min stimulation with EGF (10 ng/ml); and 4) SF medium supplemented with the RSK inhibitor BI-D1870 (3 μM) overnight, followed by a 15 min stimulation with EGF (10 ng/ml). Moreover, to test RSK2 activation, HeLa cells overexpressing or not RSK2WT, RSK2S227D, RSK2Y707A, RSK2S227D/Y707A or RSK2K100A were cultured in serum free medium overnight, and then treated or not with phorbol myristate acetate (PMA, 60 ng/ml) or EGF (10 ng/ml) for 5 minutes. Finally, to test and compare RSK1 and RSK2 mutants’ activation, HeLa cells overexpressing GST, GST-RSK1WT, GST-RSK1S221D, GST-RSK1K210A, GST-RSK1R108A, GST-RSK2WT, GST-RSK2S227D, GST-RSK2K216A, GST-RSK2R114A, GST-RSK2K100A and GST-RSK2Y707A were cultured in normal culture medium overnight and, then, starved for 3 h and treated with EGF (10 ng/ml for 15 min). After treatment, cells were washed twice with PBS 1X and lysed with 300 µl of “Lysis Buffer” (0.5 M Tris-HCl pH 6.8, 10% SDS, 10% Glycerol). Lysates were then sonicated for 10 sec, in order to break DNA strand and to reduce viscosity. After that, samples were heated at 95 °C for 5 min. 10 µl each sample were used for BCA quantification at 562 nm wavelength and variable volumes (corresponding to 5 or 10 µg of proteins), were loaded in 7.5% or 4–15% SDS-PAGE wells. Proteins were run in polyacrylamide gel at constant voltage (200 V) for 35 min. Then, proteins were transferred on a PVDF membrane, using Trans-Blot Turbo BIORAD. The PVDF membranes were let dry, and then hydrated with TBS (Tris Buffer Saline) 0.1% Tween for 30 min at room temperature (RT). Afterwards, membranes were incubated with primary antibody (Ab) under gentle agitation overnight at 4 °C. Monoclonal Ab rabbit anti-pT359RSK, monoclonal Ab rabbit anti-pS380RSK, monoclonal Ab rabbit anti-pS102YB1 and monoclonal Ab rabbit anti-YB1 were purchased from Cell Signaling Technology. Polyclonal Ab rabbit anti-pS221/227RSK1/2 was purchase from Thermo Fisher Scientific. Polyclonal Ab mouse anti-RSK2, polyclonal Ab rabbit anti-RSK1 and polyclonal Ab goat anti-β-actin were purchase from Santa Cruz Biotechnology. Each antibody was diluted 1:1000 in TBS, 0.1% Tween, 5% BSA. After primary Ab incubation, the membrane was washed 3 times with TBS 0.1% Tween, then incubated at RT under gentle agitation with secondary Ab (anti-mouse, anti-rabbit or anti-goat), which are conjugated to Horseradish Peroxidase (HRP) enzyme. The secondary antibody was used at 1:10000 dilution in TBS 0.1% Tween. After secondary Ab incubation, the membrane was washed 3 times with TBS 0.1% Tween, and incubated for 1 min in the Western Lightning TM Chemiluminescent Reagent Plus ECL (PerkinElmer), containing H_2_O_2_ and luminol. The peroxidase linked to secondary antibody oxidizes, in the presence of H_2_O_2_, the luminol, which decays from an excited state, generating light. Generated light is used to impress a photographic film. Loading control was performed using β-actin immunodetection.

### Pull-down and kinase assay

pDEST27-RSK2WT and pDEST27-RSK2S227D plasmids were transfected in 293T cells with calcium phosphate (Promega). Then, cells were treated or not with the PDK1 inhibitor GSK2334470 (3 µM). After 16 h, cell lysates were extracted in ice using cold lysis buffer (25 mM Hepes, 300 mM NaCl_2_, 1 mM PMSF, 1.5 mM MgCl_2_, 0.5% Triton X-100, 20 mM Na-β-glycerophosphate, 1 mM Na_3_VO_4_, 0.2 mM EDTA, and 1:1000 protease inhibitor cocktail; Sigma-Aldrich). GST-tagged proteins were isolated through Glutathione Sepharose 4B beads (GE Healthcare). The GST-tagged proteins isolated from 293T bound to glutathione beads were used to pull-down proteins from 293T extracts and they were washed with the same buffer used before but containing 1 M NaCl_2_. The pulled-down proteins were dissociated using a reducing buffer (10mM L-Glutathione Reduced and 50 mM Tris-HCl, ph 8.0) and analyzed by immunoblot. RSK kinase assay was performed by using Abcam p70 S6K Activity Kit (ab139438), which is based on a solid phase enzyme-linked immuno-absorbent assay (ELISA) that utilizes a specific synthetic peptide as a substrate for p70-S6K, but also for p90-S6K (RSK), and a polyclonal antibody that recognizes the phosphorylated form of the substrate. Purified GST-RSK2WT and GST-RSK2S227D were diluted 1:30 in reducing buffer (10 mM L-Glutathione Reduced and 50 mM Tris-HCl, ph 8.0). Then, Kinase Assay Dilution Buffer was added to samples, as reported in the assay procedure, and transferred in appropriate wells of the kit plate. To initiate the kinase reaction 0.5 mM ATP was added in each well and the plate was incubated at 30 °C for 1 h. Next steps were performed as described in the assay procedure. Finally, the reaction was stopped and absorbance of each sample was measured at the wavelength of 450 nm.

### Molecular dynamics (MD) simulation

The crystal structure of N-terminal RSK2 kinase in complex with 2-amino-7-substituted benzoxazole (PDB ID 4NW5) was taken into account since it shows the lowest *RMSD* value (0.954 Å) compared to active Akt/PKB (PDB ID 1O6K). Moreover, the loop carrying Ser227 was phosphorylated and visible in this structure. The Ser227 was mutated into Asp (Asp227) to obtain the phosphomimetic, using UCSF Chimera software. The most favorable rotamer for Asp227 was considered. The Mg-ATP bound state was generated by using the coordinates of Mn2AMP-PNP from the structure of Akt/PKB complexed with a GSK3 peptide (PDBID 1O6K).

A 20 ns MD simulation was run on the protein containing pSer227 and on the molecule carrying S227D mutation using the Yasara package^58^ and AMBER14 force field at 1 atm, pH 7.4 and 298 K of temperature. The simulations were set up with 1.25 fs time step under periodic boundary conditions. The timescale of the molecular dynamics simulations (20 ns) was chosen to see the local structural effects of the substitution of pSer to Asp, especially for what concerns the salt bridges network. Such timescale however prevented us to simulate large movements of the kinase lobes, that happen in the order of microseconds and that would require a much larger computing power, not available with the current technology^59,60^.

### Statistical analysis

All the data are represented as the mean value of at least three experimental replicates and error bars represent standard deviations. Statistical analysis was performed using GraphPad Prism software (GraphPad Software). Statistical significance was determined by a Student’s t test with P < 0.05 considered significant.

## Supplementary information

Supplementary material.
