# Supplementary material for: Activation of RSK by phosphomimetic substitution in the activation loop is prevented by structural constraints
Source: Sci Rep. 2020 Jan 17;10:591. doi: 10.1038/s41598-019-56937-3 (PMC6969211; doi:10.1038/s41598-019-56937-3)
Supplement: Supplementary file 1 — Supplementary material. [file 41598_2019_56937_MOESM1_ESM.pdf]

Activation of RSK by phosphomimetic substitution in the activation loop is prevented by structural constraints

Desiana Somale[1,2], Giovanna Di Nardo[3,4], Laura di Blasio[2], Alberto Puliafito[1,2], Marianela Vara-Messler[1,2], Giulia Chiaverina[1,2], Miriam Palmiero[1,2], Valentina Monica[1,2], Gianfranco Gilardi[3,4], Luca Primo[1,2,*], Paolo Armando Gagliardi[2,*]

1. Department of Oncology, University of Torino, Turin, 10060, Italy
2. Candiolo Cancer Institute-FPO IRCCS, Candiolo, 10060, Italy
3. Department of Life Sciences and Systems Biology, University of Torino, Via Accademia Albertina 13, Torino, Italy.
4. CrisDi, Interdepartmental Center for Crystallography, University of Torino, Via Pietro Giuria 7, Torino, Italy

* These authors contributed equally to the work

Fig. S1

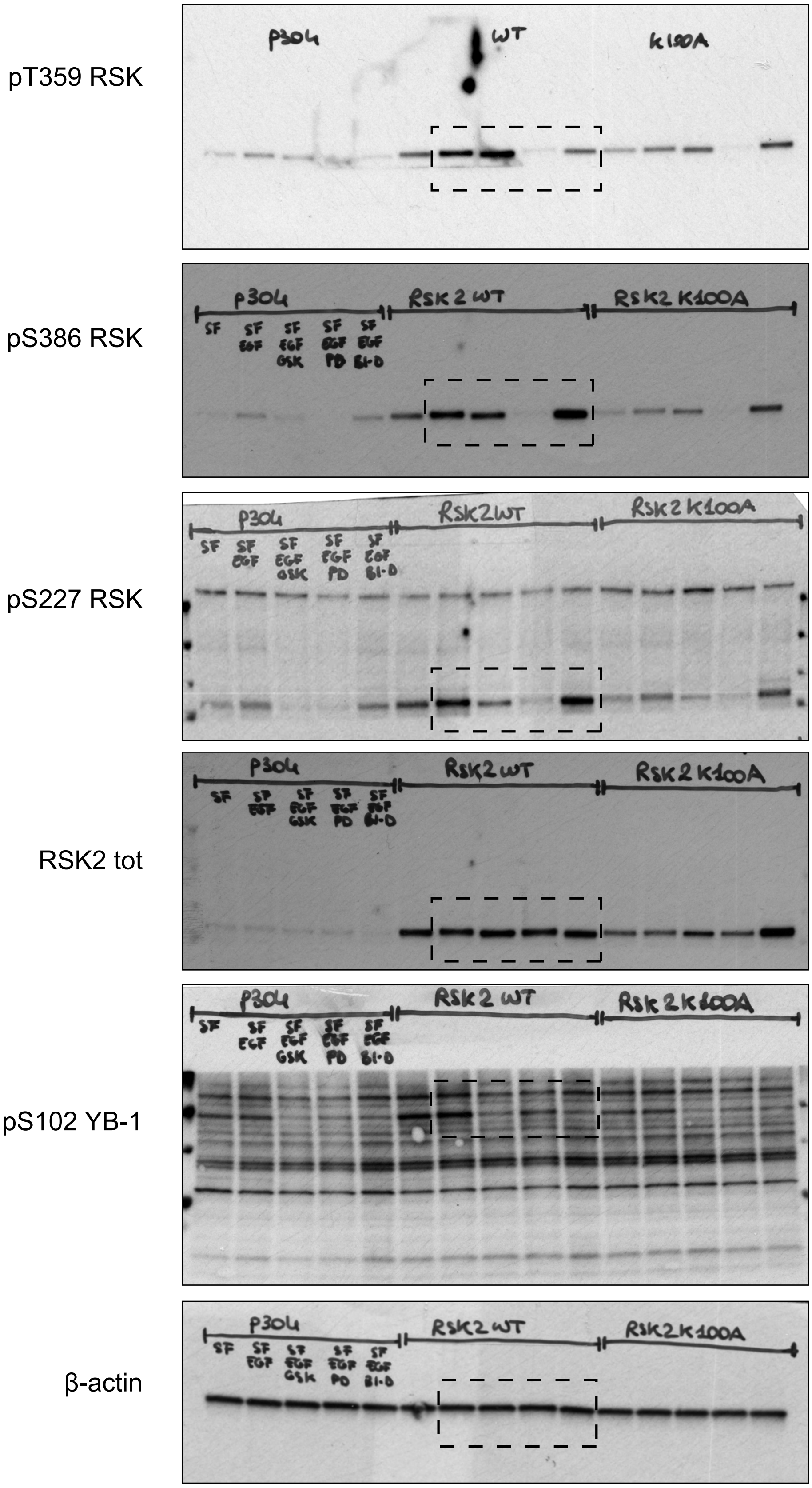

Figure S1: Unprocessed western blots relative to figure 2A.

The original western blots are part of a larger experiment in which we compared wild type, RSK2 WT and RSK2 K100A overexpressing cells. However, to avoid redundancy we reported in figure 2A only the lanes corresponding to RSK2 overexpressing cells in presence of EGF, as described in the legend of figure 2. The cropped regions shown in figure 2A are highlighted with dashed boxes.

Fig. S2

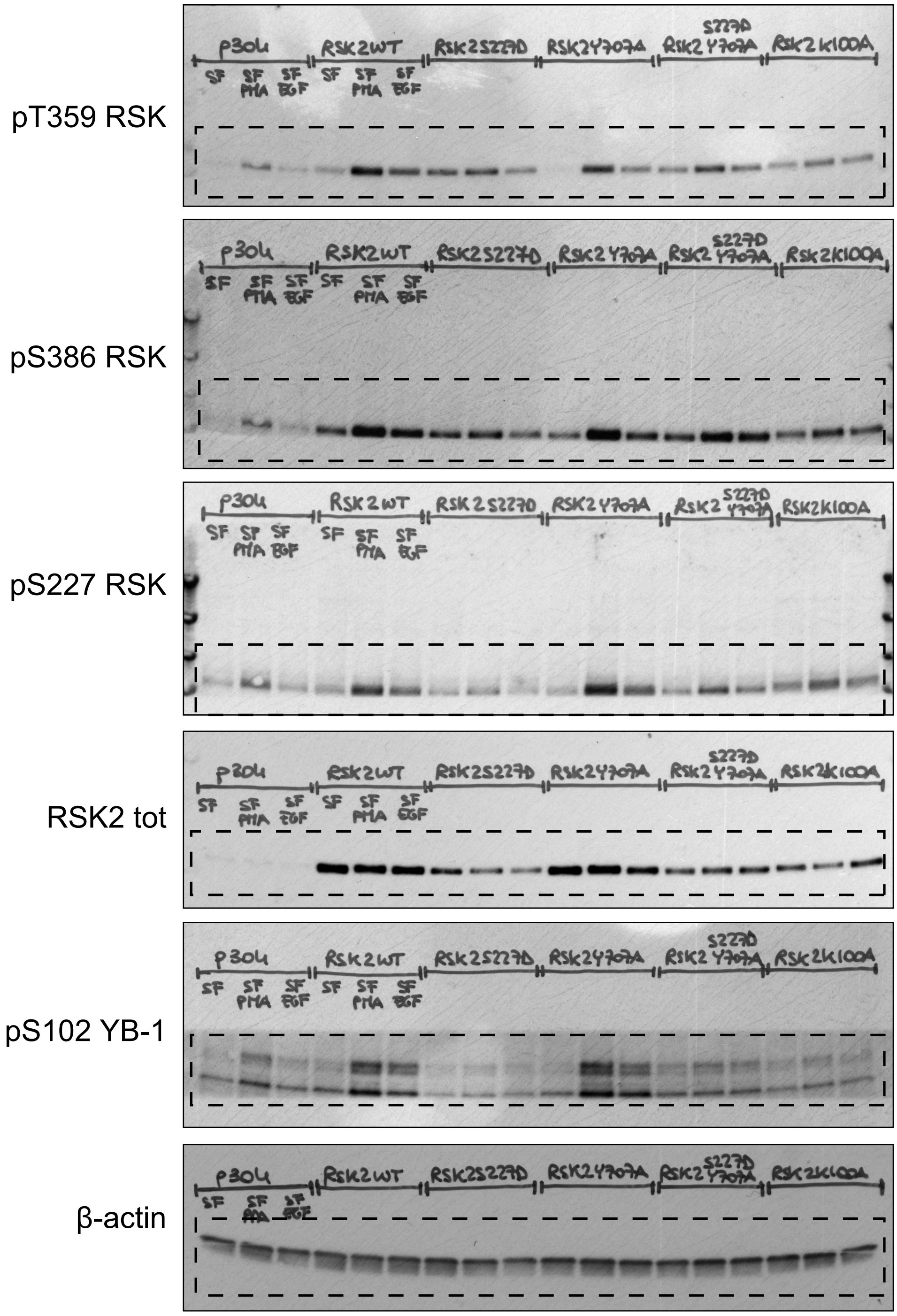

Figure S2: Unprocessed western blots relative to figure 2D.

The cropped regions shown in figure 2D are highlighted with dashed boxes.

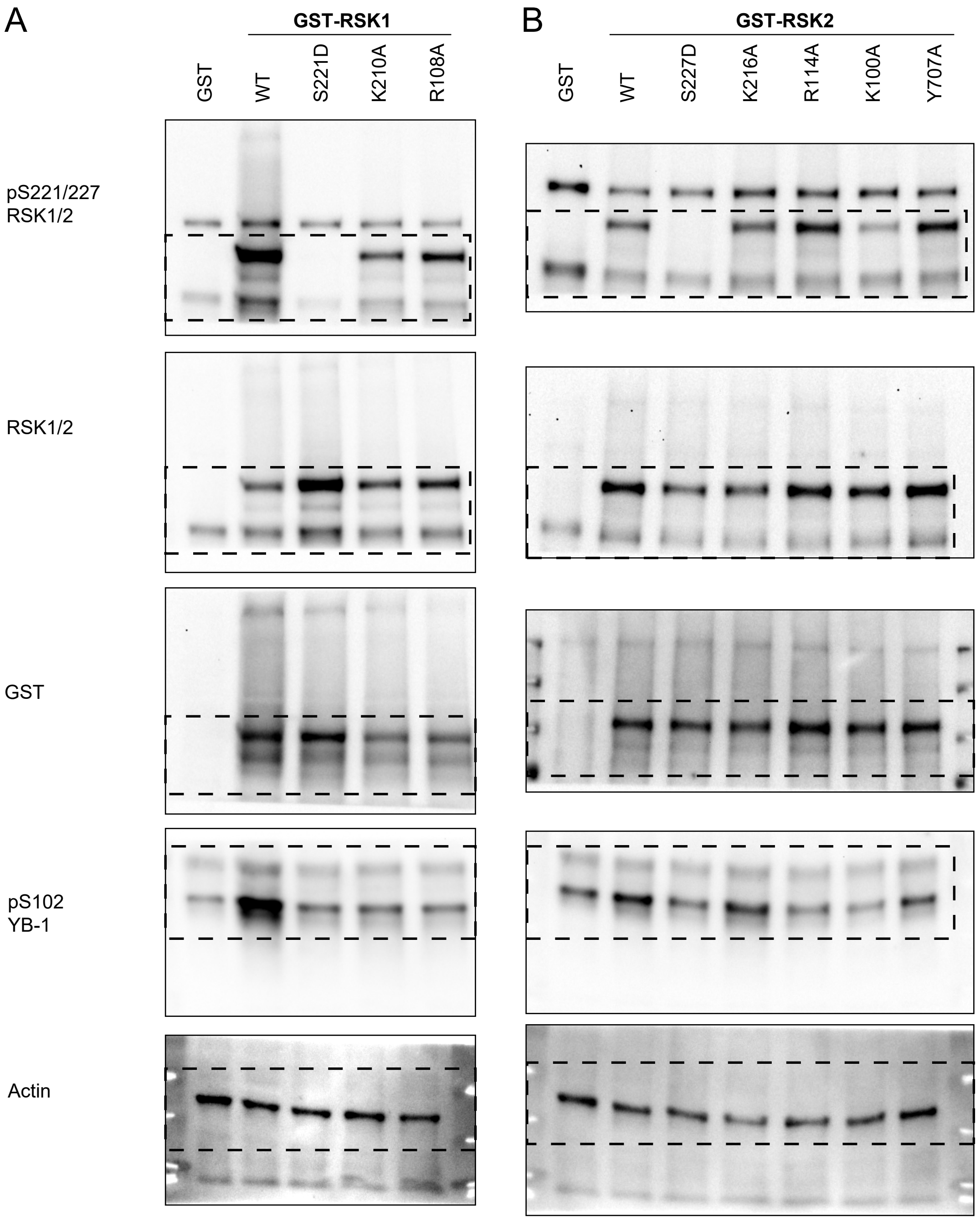

Figure S3: Unprocessed western blots relative to figures 5A and 5B.

Panel A reports the unprocessed blots shown in figure 5A, whereas panel B reports the unprocessed blots shown in figure 5B. In both cases, the dashed boxes highlight the regions shown in the main figures.
